# Improving the practice of cataract surgical outcome measurement

**Published:** 2019-02-10

**Authors:** Nathan Congdon, Sarity Dodson, Ving Fai Chan, Wanjiku Mathenge, Elise Moo

**Affiliations:** 1 Ulverscroft Chair of Global Eye Health: Queens University Belfast & Orbis International, Royal Victoria Hospital, Belfast, Ireland, UK.; 2 Global Lead – Development Effectiveness: The Fred Hollows Foundation, Sydney, Australia.; 3 Research Manager: Brien Holden Vision Institute, Durban, South Africa.; 4 Consultant Ophthalmologist and Director of Training and Research: Rwanda National Institute of Ophthalmology and Dr Agarwal's Eye Hospital, Kigali, Rwanda.; 5 Global Research Coordinator: The Fred Hollows Foundation, Sydney, Australia.

**A successful cataract outcome monitoring and continuous quality improvement system will assist practitioners and centres to identify and implement ongoing improvements in eye care delivery.**

**Figure:**
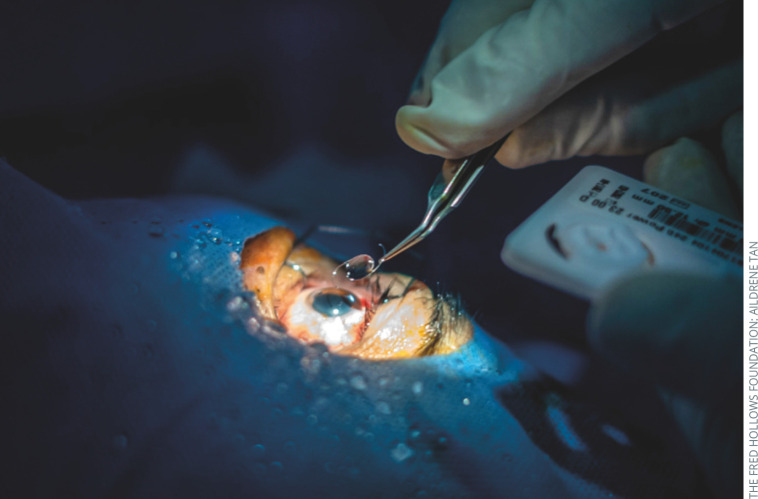
Monitoring surgical quality allows clinicians and administrators to identify issues and take action to improve practice, outcomes and performance.

A study from Kenya^1^ showed that monitoring the visual outcomes of cataract surgery is associated with improving those outcomes. This suggests that we need to know how well our patients see after surgery to have the motivation and information to improve surgical results. Tools to help with this monitoring process, both paper-based and computer-based, have been developed and made freely available.^2^ However, the practice of monitoring outcomes has not yet become a routine part of running ophthalmic services other than in situations where it is demanded by regulatory authorities or funding agencies.

## Why does measurement of cataract surgical quality matter?

Despite the availability of effective and inexpensive surgery, unoperated cataract is responsible for 35% of global blindness.^3^ The cataract blindness problem is further worsened by poor surgical outcomes, particularly in low resource settings.^4^

Quality of surgery and resulting patient satisfaction are the engines that drive sustainable cataract services. Monitoring surgical quality allows clinicians and healthcare administrators to identify issues and take action to improve practice, patient outcomes and centre performance because “if you measure it, you can manage it.” Outcome reporting, however, varies widely across surgical centres internationally^5^ and many countries where data is available fall well below World Health Organiz ation (WHO) standards for cataract surgical outcomes.^6^

## Why don't we routinely measure cataract surgical outcomes?

There are several challenges associated with routine measurement of cataract outcomes, including:

- Pressure on clinicians to generate high volume of surgical outputs
- Weak culture of quality assurance in surgical centres
- Low access to systems and tools to support continuous quality improvement
- Concern about management of complex cases
- Low rates of patient follow-up, because of the challenges getting patients to return to surgical sites several weeks following their procedure.^7^

However, these issues can be addressed by setting up a good cataract outcome monitoring and continuous quality improvement (CQI) system. This can assist practitioners and centres to identify and implement ongoing improvements in eye care delivery.

## What needs to be in place?

The essential elements of a successful outcome monitoring and continuous quality improvement (CQI) system that can assist practitioners and centres to identify and implement ongoing improvements in eye care delivery are described below.

### 1 Quality standards

Defining a ‘good’ outcome, especially with modern small-incision surgery, is the foundation of an effective CQI system. The World Health Organization (WHO) provides standards for postoperative acuity at 6 weeks^6^ (see Table 1).

**Table 1:** Standards for postoperative visual acuity

	PRECOG standards for postoperative assessment (1–3 days after surgery)	WHO standards for postoperative assessment (6 weeks after surgery)
Good (6/6-6/18)	>60%	>80%
Borderline (<6/18-6/60)	<35%	<15%
Poor (<6/60)	<5%	<5%

The PRECOG study^7^ has demonstrated that visual acuity results the day after surgery are highly predictive of final vision. Outcomes can, therefore, also be measured 1 to 3 days after surgery. This measure of the quality of surgical outcomes is equivalent to WHO standards, but may be more convenient for many surgeons and patients, particularly in areas with low postoperative follow-up rates, (e.g., when patients have to travel long distances).

Table 1 shows the PRECOG standards for postoperative assessment 1–3 days after surgery alongside WHO standards for postoperative assessment 6 weeks after surgery.

### 2 Timely and routine data capture

Effective, accessible and easy-to-use data collection tools, whether electronic or on paper, improve data quality and reduce the burden of monitoring activities on clinicians and administrators.

**Figure 1:**
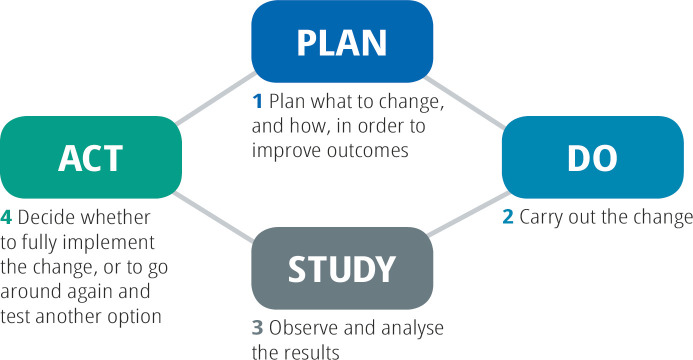
The Plan-Do-Study-Act model of continuous quality improvement

### 3 Accessible reports

Simple, visual reports of key results encourage surgeons and administrators to engage meaningfully with outcome data.

### 4 Feedback and interpretation of results

Interpret the results and give supportive, non-blaming feedback to surgeons. This is an opportunity to identify potential corrective actions.

### 5 Ongoing improvement processes

Ongoing improvement processes make up the critical final element. High quality data and ideas regarding practice and system change can only improve outcomes if they are acted upon.

**The BOOST cataract app**

BOOST (Better Operative Outcomes Software Tool) is a free Android and online app that allows surgeons to easily capture key cataract outcome data. It provides simple, engaging reports and provides feedback to users about how they can improve their performance.

BOOST is an international effort, and is available in seven different languages: English, French, Spanish, Russian, Chinese, Vietnamese and Bahasa Indonesia.

It can be downloaded at the Google Play Store by searching for ‘BOOST Cataract’, and data can be accessed online at https://boostcataract.org/

BOOST takes users through two simple steps designed to evaluate and improve cataract surgical results.

**Step 1 Benchmarking**

Using BOOST, surgeons enter the uncorrected visual acuity on postoperative day 1 for 60 consecutive operated eyes. The app then tells surgeons how their results compare (anonymously) with other users globally.

**Step 2 Quality improvement**

The BOOST app then asks users to record the results of 20 consecutive patients with poor results (≤ 6/60) at least 6 weeks after surgery. In each case, the user is prompted to choose one of three reasons for the poor result:

- Surgical complication
- The presence of another blinding condition (such as glaucoma, ARMD, DR, etc.)
- Refractive problems (wrong power IOL, lack of any IOL, etc.)

The app determines what the most common cause of poor vision is for any given user, and then makes specific recommendations to improve outcomes. For example, if the most common cause is the presence of other diseases, the app recommends specific ways to address this problem, such as a thorough preoperative examination of the eye, including the fundus, with dilation of the pupil after checking for an afferent pupillary defect.

**Figure:**
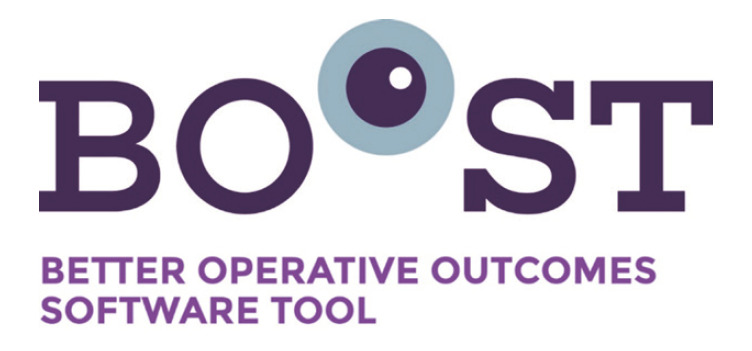

**Tips for using the app**

To get the most out of the app, you need to be honest with yourself about the data you enter. Remember two things:

1. Enter data from consecutive patients, which means you need to enter every cataract case you do (no combined cases or paediatric cataract cases), unless the patient is known to have a problem in addition to cataract.
2. Vision should be measured and entered by someone other than the operative surgeon.
